# Cyclopædiæ of Medicine and Surgery

**Published:** 1840-04-01

**Authors:** 


### CYCLOPÆDIÆ OF MEDICINE AND SURGERY.

I. A DICTIONARY OF PRACTICAL MEDICINE: Comprising General Pathology, the Nature and Treatment of Diseases, Morbid Structures, and the Disorders especially incidental to Climates, to the Sex, and to the different Epochs of Life; with numerous Prescriptions for the Medicines recommended, a Classification of Diseases according to pathological principles, a copious Bibliography, with references; and an Appendix of approved Formulæ: the whole forming a Library of Pathology and Practical Medicine, and a Digest of Medical Literature. By *James Copland*, M.D. F.R.S. &c. &c. Parts V. and VI. London, Longman, and Co.

II. THE CYCLOPÆDIA OF PRACTICAL SURGERY, EMBRACING A COMPLETE VIEW OF ALL THE DEPARTMENTS IN OPERATIVE MEDICINE. Edited by *William B. Costello*, M.D., Member of several Learned Societies, National and Foreign. Part IV. London, Sherwood and Co.

WE cannot but look on the several Cyclopædiæ that have lately been or are at this time in process of publication, as at once the indices and agents of the march of improvement in medicine. When we compare their copious pages with the meagre ones of dictionaries not yet extinct, we are struck at once with the difference. This obtains more particularly in the morbid anatomy of diseases, and in the consequent precision with which symptoms are defined and treatment regulated.

Amongst the Cyclopædiæ, that of Dr. Copland, though modestly designated a dictionary by its author, holds, we think, the first rank. In completeness it has not suffered by being confided to a single hand, and, in unity, it has gained.

The last two Parts of the "Dictionary" lie before us. They embrace the consideration of many of the Diseases of the Heart—Herpetic Eruptions—Hiccup—Hooping Cough—Hydatid—Hypertrophy—Hypochondriasis—Hysteric Affections—Jaundice—Ichthyosis—Impetiginous Affections—Impotence and Sterility—Impotence in the Male—Impotence and Sterility in the Female—Indigestion—Induration—Infection—Inflammation—Influenza—Insanity. The latter subject is not quite completed.

Although we cannot notice these Parts in detail, we can speak of them as satisfactorily as if we analysed them. They are equal to their predecessors, and sustain the reputation, a high and a well-earned one, of Dr. Copland. This will be universally admitted to be no trifling praise.

We shall select a few of our author's opinions on some practical and important points, or a few striking facts. It would be useless to do more.

1. *Danger of large Blood-letting in Hypertrophy.*

We quite coincide with Dr. Copland in reprehending those large bleedings for hypertrophy of the heart which some physicians have recommended. After quoting and dissenting from such recommendations by Laennec and by Bouillaud, Dr. Copland observes:—

"Respecting *bloodletting* in this malady, my experience and opinions are in accordance with those of Dr. Hope; and I consider, with him, sparing abstractions of blood, at intervals of two or three weeks or more, to be the most beneficial. More copious depletions have given temporary relief; but the symptoms have soon returned with increased violence, and carried off the patient, especially in cases where there were also dilatation and lesions of the valves or orifices of the heart. As I have shown in the article Blood, large depletions increase the frequency of the heart's action; and this effect is more readily produced by them when this organ is in a state of enlargement. I perfectly agree with the above writer in considering, that the indications of treatment should be to diminish the quantity, without deteriorating the quality, of the blood, and without producing reaction, or permanently enfeebling the action of the heart and the energies of the constitution,—that from four to eight ounces of blood, taken every two, three, four, or six weeks, according to the circumstances of the case, will be sufficient to fulfil this indication, to keep down inordinate action, and to relieve the dyspnœa,—that the diet should be spare, and consist of white animal food, and liquids in small quantity; and that everything heating or stimulating, or calculated to accelerate the circulation, ought to be avoided." 209.

This is judicious advice. The great thing in the treatment of diseases of the heart is to avoid all sources of vascular or nervous excitement—to maintain the secretions, more particularly of the kidneys—to prevent congestions of the vascular system by leeches or cupping or gentle bleeding—and to tranquillize by sedative applications or medicines.

2. *Treatment of Herpes.*

We are induced to allude to this, because it is often troublesome and, medical practitioners frequently experience more embarrassment in cutaneous than in other and serious disorders.

"Keeping," says Dr. Copland, "the connection of the eruption with disorder of the digestive organs closely in view, a mild ipecacuanha emetic should be exhibited, and subsequently any gentle purgative, with magnesia or an alkaline carbonate. Afterwards a free use of diluents and abstinence are all that will be required in most cases. In the more severe attacks, especially of herpes zoster, additional means will often be called for. Where there is much antecedent or attendant fever, M. Rayer advises a moderate bleeding, or the application of a number of leeches to the anus or around the seat of eruption. Neither of these is often necessary. When the evacuations are morbid, and the biliary functions impaired, a dose of blue-pill or of calomel, at bed-time, and a mild purgative, containing an antacid, the following morning, will generally be of service. It may be even requisite to repeat these; and afterwards, particularly when the urinary and fæcal excretions are disordered, to promote the actions of the liver and kidneys, by small doses of colchicum with magnesia or an alkaline subcarbonate. In the more painful cases of zona, these means will be found most beneficial. During the course of the complaint, the diet should be mild, chiefly farinaceous, and in small quantity. The beverages should be demulcent and cooling." 233.

We should say, from our experience of Herpes, that, in persons of pretty strong constitution, smart purgatives, of a mercurial character, and combined with alkalies and saline diuretics, are the staple articles of treatment. Nor is the warm bath, that important item in the management of so many cutaneous complaints, to be neglected.

"When herpes assumes a chronic character, owing to the successive eruption of clusters of vesicles, or to the excoriation of the inflamed skin, small doses of blue pill, or of the hydrargyrum cum creta, and mild stomachic aperients, are the most appropriate means. In addition to these, the decoction of sarsa, or of the elm-bark, with liquor potassæ, are often very serviceable. In *herpes labialis,* and *herpes præputialis,* these remedies are especially required. In more obstinate cases, particularly when the excretions continue disordered, mild stomachic purgatives, and alteratives, should be persisted in; and warm or tepid bathing, or even vapour baths, occasionally employed. In *herpes iris,* the warm bath, and minute doses of the arsenical solution with the liquor potassæ, are generally of service. Dr. A. T. Thomson, recommends, for this species, the decoction of the Rumex obtusifolius with these alteratives." 233.

We would observe that the vesicular eruptions which become chronic, and are characterised by either considerable irritation of the surface, or by much discharge, have always appeared to us to be particularly benefited by active aperients of a mercurial character, and by the liquor potassæ pushed to full doses. Whether the latter acts mainly by its diuretic or its other properties we have never been able to determine satisfactorily. But that it *is* very serviceable we are well assured. Many persons seem to us to give the liquor potassæ as a matter of course in cutaneous disorders. So far as we have seen, it proves most serviceable under the circumstances we have mentioned.

"When herpes occurs in cachectic or aged persons, not only should great attention be paid to the state of the excretions, all fæcal and morbid accumulations being duly evacuated; but the digestive and assimilating functions ought to be promoted, by exhibiting gentle tonics, with the alkaline carbonates. If the eruption ulcerate, the application of nitrate of silver, in substance, or in a strong solution, will promote cicatrisation. If there appear a disposition to slough, the preparations of bark, &c. will be required. When violent sub-cutaneous pains accompany zona, hyoscyamus or other narcotics may be given with the medicines already recommended; but the warm or vapour bath, and colchicum, as above prescribed, will be found the most successful. In *herpes præputialis*

and *herpes vulvæ*, the early application of nitrate of silver will often shorten the duration of the eruption. Where there are much heat and stinging of the parts, a wash containing the subborate of soda, or the sulphate of zinc, or of alumina, will often be useful. These may also be prescribed in herpes circinnatus; but in all cases the chief attention must be directed to the removal of disorder in the digestive and biliary organs, and to the regimen of the patient." 234.

Of all tonics the infusion of sarsaparilla, with or without alkalies, has, in our hands, answered best. We would observe that we have twice seen a very troublesome sore succeed the application of the nitrate of silver to herpes præputii. We believe that the best thing for it is a lotion of the acetate of lead or of the sulphate of zinc. Some individuals with a long prepuce are extremely prone to the affection.

### 3. *Treatment of Hiccup.*

Hiccup depends on many causes, and to relieve it they must, of course, be investigated, and the remedy suited to the particular nature of the case. But idiopathic hiccup, unconnected with any obvious lesion or appreciable disturbance of the function of any organ, is occasionally a troublesome disorder. Dr. Copland remarks that, in this form, the administration of *opium* with *ether*, or of other *anodynes* and diffusible *stimulants*, and of refreshing alkaline beverages, will generally give relief. Various *antispasmodics*, volatile nervines, and sedatives, especially *camphor*, *ammonia*, *hyoscyamus*, *hydrocyanic acid*, either taken into the stomach, or inhaled into the lungs with warm vapour, will often remove the complaint. Idiopathic hiccup also may cease spontaneously; or it may be arrested by exciting some powerful mental emotion, as surprise, fright, &c., or by powerfully exciting the diaphragm by *sternutatories* or *emetics*; or by taking any substance in quantity into the stomach.

In obscure or doubtful cases, *camphor*, with or without the *nitrate of potash*; the *spiritus ætheris nitrici*, or the *spiritus ætheris sulphurici comp.*, or the *tinctura camphoræ composita* may be given with *demulcents*. The alkaline *sub-carbonates* may also be exhibited with *hyoscyamus*, or with *colchicum*, *opium*; or the *hydrocyanic acid* may be given in an aromatic or gently tonic infusion.

### 4. *Classification of Infectious Agents.*

In a very elaborate and very useful article on infection, Dr. Copland presents us with a Table, embodying in one group the various infections. We think it worth presenting.—*(See following page.)*

It is not to be supposed that such a thing as a Classification of Infections could be executed without opening a door to doubt and criticism. We may therefore observe, without fear of giving offence, that it is not quite certain that fevers which have their source, or are supposed to have their source, in vegetable miasms, or at least in effluvia from marshes, &c., never subsequently spread by infection; nor do we esteem it quite proved that pestilential cholera or even yellow fever, is to be put in the same category with typhus. Yet the classification, bating some disputable points, is comprehensive and accurate.

| Class of Agents. | Order of Agents. | Species of Agents. | Diseases resulting therefrom. |
|---|---|---|---|
| I. NON-DISSEMINATING, AND NON-PERPETUATING INFECTIONS. *Idio-infectants.* | i. *Miasms or mephitic vapours—Endemic Infection—acting through the air.* | 1. Miasms from decayed vegetable matter aided by moisture, in temperate ranges of atmospheric heat. | Catarrhal fevers. Rheumatic attacks. Intermittents. Enlargements of the spleen, and torpid states of the liver. |
| | | 2. Exhalations from absorbent, or deep, exhuberant, or marshy soils, suspended in atmospheric humidity at temperate grades of warmth. | Intermittents. Remittents. Simple dysentery. Simple cholera. Bilious fevers. Obstructions and other diseases of the liver and glandular organs. |
| | | 3. Miasms or vapours from decayed vegetable matter, or from marshes and rich, deep, and humid soils, at high ranges of temperature. | Inflammatory, bilious and gastric fevers of both a remittent and continued type. Diseases chiefly of the abdominal viscera. |
| | ii. *Unwholesome and poisonous ingesta*—Infections occasionally epidemic. | Unripe, diseased, or decayed grain. Diseased or putrid fish or flesh. Water containing putrid animal matters, &c. &c. &c. | Ergotism. Gangrenous ergotism. Asthenic and chronic diarrhœa. Dysentery. Scurvy and scorbutic dysentery. Mucous, gastric and putro-adynamic fevers. |
| | iii. *Self contaminating agents,* or morbid matters formed in a part, afterwards contaminating the system generally. | 1. Cancer. Fungo-hæmatoid disease, &c. | The carcinomatous and fungo-hæmatoid cachexy. |
| | | 2. Purulent, sanious, or other morbid secretions carried into the circulation. | Acute hectic. Low remittent, and adynamic states of fever, often attended by phlebitis or purulent deposits in the viscera or joints. |
| INFECTIONS.— | i. *Animal effluvia.*—Producing diseases propagating the same or similar maladies in favourable circumstances. | 1. Effluvia from animal matter or from vegeto-animal matters during decomposition, aided by humidity. | Adynamic or pernicious remittents. Continued fevers. Adynamic dysentery. Cholera. Gastric, mucous, or enteric fevers. |
| | Conditionally and consecu- | 2. Emanations from living bodies in close or unventilated situations. | Adynamic putro-adynamic, and malignant fevers. Malignant dysentery. |

| | | | |
|---|---|---|---|
| II. CONDITIONALLY PERPETUATING *Contaminating Infections.* | *tively infectious, chiefly by* means of diffusive and impalpable emanations. | 3. Emanations from the secretions and discharges of the sick confined in close apartments, &c. and the direct application of these secretions. | Erysipelas. Hospital gangrene. Phlebitis. Puerperal fever. |
| | ii. *Animal secretions and septic animal matters.*—Infectious chiefly by contact, or inoculation of a palpable matter; *chiefly sporadic.* | 1. Morbid secretions in recently dead bodies. | The irritative fever, or malignant effects produced upon opening recent bodies, by the morbid secretions poured out in serous cavities. |
| | | 2. Animal matter in a state of putridity or decomposition. | Diffusive or disorganising inflammation of cellular parts. Inflammation of lymphatics of veins, &c. |
| | | 3. Morbid secretions communicated from the lower animals by contact or inoculation. | Glanders. Farcy, malignant pustule and other affections arising from contaminating diseases in the lower animals. |
| | | 4. The poisonous bites of insects and reptiles. | General vital depression, and septic disorganization, or solution of the vital cohesion of the tissues. |
| III. SPECIFIC INFECTIONS.—Infections immediately or mediately disseminating and perpetuating their kinds, by a morbid impression, or by contamination, or by both. Capable of retention and communication by Fomites. | i. *Impalpable specific infections* of susceptible persons. Diffusive or volatile infections, frequently epidemic. | Emanations from the secretions, excretions, and surfaces of persons already affected.—Propagating their kind by a diffused and impalpable effluvium, or vapour. | Epidemic and exanthematic typhus. True yellow fever. Pestilential cholera. Pertussis. |
| | ii. *Palpable specific contagions.* Consistent contagions. | A specific secretion or virus from the seat of disease perpetuating maladies always presenting the same characters. | Rabies. Syphilis. Gonorrhœa. Scabies. Yaws. Sivvens. Frambœsia. Purulent or Egyptian Ophthalmia. Cow-pox. Pellagra. Porrigo. |
| | iii. *Infections both diffusive and consistent.*—Often epidemic. | Diffusive and impalpable emanations, and consistent secretions, from the bodies of the infected, either of which may produce the same disease. | Chicken-pox. Scarlet fever. Small-pox. Measles. Malignant puerperal fever. Plague. |

5. *The Union Workhouses and Typhus.*

Dr. Copland makes some observations which we do not scruple to bring forward. If founded in truth, they ought to stir up inquiry and draw down retribution on the heads of the guilty—if in error, those criminated should gladly exculpate themselves.

" I am convinced that the low dietaries assigned to the poor in the Union workhouses, in connection with crowding, and with imperfect ventilation, in many of them, have been a chief cause of the present prevalence of typhus throughout the country. And although the infectious visitation may not have reached those who have been the prime movers in the iniquity, yet it may overtake some of them with no measured retribution. Persons who require the aid of the Poor Law have usually, as respects food or drink, and sometimes as regards both, lived fully or intemperately; and when they are subjected to a diet, altogether insufficient for the continuance of health even in the temperate, low fever, which readily propagates its kind among the predisposed, and on occasions favouring communication, soon makes its appearance. This result the more certainly follows, when numbers are similarly circumstanced, and placed in buildings possessing no thorough ventilation or perflation of air. The only recently erected Union workhouses which I have seen, are most improperly planned, inasmuch as they have windows only looking into the interior of the Court, of which they form three of the sides. This is shameful if it proceed from ignorance, and flagitious if it be done designedly. We can hardly suppose architects so ignorant of the most generally acknowledged principles of their art, as to neglect ventilation where it is most required. Are we therefore to consider that they have been controlled by those who have sacrificed feelings of humanity to the expediency of political economy." 348.

We do not conceive that political economy is answerable for such inhumanity. Some political economists may be so, but the crime, if it exist, is the peculiar and private property of selfish and brutal economists.

*Treatment of Persons in whom Symptoms of Infection have appeared.*

Even when symptoms present themselves, which render it pretty certain that the patient is actually infected, Dr. Copland believes that the disease may be yet mitigated or prevented by treatment. He excepts, however, small-pox, scarlet fever, and measles.

The principles on which Dr. Copland acts are the following:—1st. That the *more immediate impression* of infections is made upon the nervous system of organic life.—2d. That this impression is of a sedative or *depressive* kind. 3d. That infectious agents not only *depress*, but also *modify* or *alter* the vital influence in a special manner, or, in other words, each infectious agent produces a peculiar or specific depressing effect.—4th. That the vascular system and circulating fluids soon experience the effects of this impression; and that the action of certain infections and contagions are earlier displayed on this system, in respect of some contagions, than as regards others.—5th. That the circulating, secreted, and excreted fluids undergo a consecutive and progressive change.—6th. That the impression on the organic nervous and vascular systems, and the consecutive changes in the fluids, ultimately affect and impair the vital constitution and cohesion of the soft solids.—7th. That, as an infectious agent exerts a depressing or sedative, as well as a special or peculiar, morbid impression, it is reasonable to infer, that whatever tends to increase the nervous power, will enable the energies of life to resist the morbid impression, to prevent the progress of contamination, and often ultimately to remove both their immediate and remote effects.

In many cases, therefore, Dr. Copland has advised a restorative and tonic treatment, together with proper prophylactic means. Frequently, he informs us, this treatment should be preceded by an *emetic*, which may be conjoined with some warm or stimulating substance. A warm *stomachic purgative* should afterwards be exhibited; and hot diluents, with camphoratad or aromatic substances, may also be given. The energies of life ought to be promoted by means suited to the habits and circumstances of the individual, particularly by tonics or light nutritious diet, and pure dry air. When the infectious agent produces, at the period of exposure to it, a sensibly depressing and morbid operation, it will be often of service to excite, as soon as possible, an artificial febrile commotion in the system, and to promote the secretions and excretions.

"When the primary operation of infectious agents is characterised by great vital depression, it is surprising how large doses of tonic and restorative substances may be taken before this state is removed. A lady of a delicate constitution, usually unable to take more than two glasses of wine after dinner, without occasioning heat and discomfort, was exposed to concentrated effluvia of the exanthematic typhus now prevalent. She felt an unpleasant odour, followed by a sudden loss of strength, nausea, and all the symptoms indicative of infection in a severe form. Her spirits were depressed; she stated her conviction that she had caught the infection, although she had approached it without any dread; gave directions as to her affairs, and resigned herself to her bed. I found her, with a weak, irregular pulse, slow and very compressible. The countenance was very pallid, and the mental and corporeal depression was extreme.—I prescribed camphor in the form of pill, and the decoction of cinchona with the compound tincture, the tincture of capsicum, the chlorate of potash, and carbonate of soda, in full and frequent doses. In the intervals, wine was given freely in the form of negus, a bottle being taken in the twenty-four hours in this form. These means were persisted in for two days, before the powers of life rallied; when a free and general perspiration broke out, and restoration quickly took place.

Of four persons who were exposed to the concentrated emanations of typhus, three of them were seen by me soon afterwards, owing to the appearance of symptoms of infection. They were all treated upon the same principles; sulphate of quinine and camphor in full doses, and as much purified extract of aloes as was necessary to keep the bowels open, were given every three or four hours, in two of these cases. The three, thus treated, soon ceased to experience the precursory symptoms. The fourth of these exposed persons was an aged female, and hence much less susceptible of typhus infection than the three young persons who had evidently caught the infection. She escaped, but carried the infection to her son and daughter." 364.

Here we shall take our leave of Dr. Copland for the present. At a future opportunity, we shall return to his valuable Dictionary, and pick out some of the many valuable things which it contains.

---

We now turn to the CYCLOPÆDIA of PRACTICAL SURGERY. A considerable delay has taken place in the production of the present Part. The articles contained in it are not of a very important character. They are, independently of some minor pieces, as follows:—Ankylosis, by W. J. Little, M.D.; Anthrax, by W. B. Costello, M.D.; Antiphlogistics, by H. T. Chapman; Anus, by John Malyn; Aqueous Humour—Aquo Membranitis, by R. Middlemore; Argenti Nitras, by H. T. Chapman; Arm, Bend of, by C. Tarral, M.D.; Arsenic, by G. G. Sigmond, M.D.; Diseases of Arteries, by W. B. Costello, M.D.; Anomalies of Arteries, by P. H. GREEN, M.B.; Traumatic Arthritis, by J. Blackburn; Articulations, by J. Blackburn; Asphyxia, by P. Bennett Lucas.

The article Ankylosis is concluded by Dr. Little in the present Part. It contains some interesting cases in which tendons were divided with great benefit. "Anus" is well handled by Mr. Malyn, who has brought together all the facts connected with a part of so great surgical importance.

The observations on "Aquo-Membranitis" by Mr. Middlemore, are interesting. Passing over his account of the symptoms, &c. we are induced to point out the treatment that he recommends.

One great point is to bear in mind, the state of health, and the constitutional vigour of the patient.

"I have subdued the severest forms of inflammation of the membrane of the aqueous humour by means of quinine, with very slight alterative doses of mercury,—mercury *not* given to the extent of producing salivation—when the disease has occurred in feeble strumous children; and this plan of management I adopted and recommended at a time when, as far as I was able to learn, it was the customary practice to treat the disease by means of mercury, as for the cure of common iritis." 355.

If the patient, though young, is tolerably strong, Mr. Middlemore advises a few leeches, and small doses of mercury so as to affect the mouth —blisters behind the ear—poppy fomentations, or an aqueous solution of opium (prepared by adding half a pint of boiling water to four scruples of powdered opium, allowing it to stand for twenty minutes, then filtering for use), or a weak goulard lotion—a mustard pediluvium every night—a darkened chamber—and a mild diet. The necessity for bleeding will be determined by the state of the parts and of the constitution.

"When extensive muddiness of the pupil is present, with merely a slight degree of inflammation, and more especially if the neural surface of the cornea is cloudy and opaque by patches, the best plan of management consists in the administration of four or five grains of the hydrargyrus c̄. cretâ every evening, with three or four grains of quinine, taken in divided doses during the day; and to these means may be advantageously added the formation of a small issue in the arm. Even in some apparently hopeless cases, where the chambers of the eye have been nearly filled with lymph, I have occasionally had an opportunity of witnessing the restoration of a very useful degree of vision, by continuing this plan of treatment, until the gums have become slightly tender; and then persevering in the use of quinine, and the occasional administration of mercury in smaller doses, until every portion of the lymph has been absorbed, except that which has become securely organized." 356.

"In the cases," he continues, after alluding to one particular instance, "of children who have been under my care, where the inflammation has been somewhat severe and protracted, and where the sulphate of quina has not been serviceable, I have prescribed, with great advantage, an evening powder, containing five grains of mercury with chalk, and three of rhubarb, and also from five to fifteen drops of liquor potassæ, to be taken twice a day. Such children have generally had a furred tongue, fætid breath, and tumid abdomen, so that, before commencing the treatment, I have thought it desirable to evacuate the bowels pretty freely with castor oil. I have tried the internal administration of iodine, but it has not answered my expectations, although it has sometimes been useful as a collyrium. A small teaspoonful of the following drops, administered in water two or three times a day, may prove serviceable in cases where the habit is sluggish, the pulse slow and languid, and where the upper lip, tips of the ears, and alæ of the nose, are in a state of hypertrophy :—

℞. Tinct. Iodinii ʒiss.

Vini Ferri ℥xivss. Misce.

In adults and elderly persons, where the symptoms of the disease have not entirely disappeared after venesection and the use of mercury, and where quinine and various mineral and vegetable tonics have been prescribed in vain, I have derived advantage from the administration of the carbonate of iron, or small doses of turpentine, and in other instances colchicum has produced a very satisfactory effect, even when it has appeared almost necessary to relinquish in despair all further attempts to relieve. The indications for the one or other of these remedial agents are afforded by the characters and peculiarities of the constitution." 356.

Mr. Middlemore objects to the operation of puncturing the cornea, the immediate effects of which he admits to be promising. In the cases in which he has performed it, all the symptoms have returned with equal and oftentimes increased severity, in twenty or thirty hours afterwards. The pain occasioned by the operation is very severe, for, in order to perform it properly, the eyeball must be steadily fixed and exposed to a pretty strong light. To do this simple operation on a healthy eye furnishes no correct guide to the trouble experienced when performing it upon an organ in a condition of acute inflammation. He gives, however, these directions for the performance of the operation, should it be deemed advisable.

The patient should be placed upon a table, as for the operation of extraction, and it is found advisable to bind up the eye upon which it is not intended to operate, as a means of steadying its fellow. A cataract-needle (sharp at the point, flattened and sharp at the sides, having a delicate groove running on that side of it opposed to the operator, when introduced with its flat side directed towards the iris, but not continued quite to its point,) is then to be passed through the cornea into the anterior chamber for a short distance, when the aqueous humour will spirt through the opening it has made. It is desirable that the instrument be so made that that part of it a little behind its point shall be so small as not quite to fill up the corneal opening, and that the point of the instrument shall not project far into the anterior chamber, lest the iris should be entangled and injured. The puncturing of the cornea does not much injure its texture, nor produce severe pain, but the unavoidable exposure of the eye to light, and the degree of compression of the eye required for the purpose of securely fixing it, occasion, in an acutely inflamed state of that organ, the most intense anguish.

Mr. Middlemore describes what he considers, and, no doubt, with reason, to be chronic inflammation of the membrane of the aqueous humour. In the instances which have occurred to him, there has been a faint blush around various parts of the cornea,—in some places extending quite to its margin, in others, terminating a little anterior to its border; a few large trunks within the sclerotica have proceeded from the periphery of the globe at its directly upper, lower, and lateral parts, forming an interrupted and faintly marked and festooned vascular zone in that situation. There has also been a nebulous state of some part of the membrane of the aqueous humour, but no definite lymph-like patches, no pain in the eye or forehead, and no intolerance of light. Sometimes the anterior part of the eye assumes an appearance of undue plenitude, but this is by no means of frequent occurrence. This state of things may continue for a long period. The treatment is simple—avoidance of exposure of the eyes to light or much exertion—counter-irritation behind the ears—hydrargyrum (Mr. Middlemore we know not on what authority calls it hydrargyrus) c̄ creta, and rhubarb, &c.

Strumous inflammation of the membrane of the aqueous humour differs but little in its local characters from those of common inflammation of this membrane. The disease is, however, less acute in its symptoms, and generally of longer duration. Mr. M. has treated it successfully with the sulphate of quinine and small doses of the hydrargyrum cum cretâ, and counter-irritation. The local applications he has found most serviceable have been a weak solution of hydriodate of potass (six grains to the half-pint of distilled water), or the goulard lotion, (prepared by adding half a drachm of the liquor plumbi acetatis to half an ounce of the wine of opium, and half-a-pint of distilled water).

All these affections are very prone to relapse, and the treatment should be continued after the appearances of inflammation are gone. Nor should the eye be used over much.

The subject of ARTERITIS is treated at some length by Mr. Costello, assisted by Mr. Porter, of Dublin. The disease is one of which so little is generally known that we shall take some notice of the present account of it.

Mr. Porter starts with admitting the difficulty of determining, either by symptoms or cadaveric appearances, the existence of arteritis. The red colouration of the lining membrane may be simply due to straining, and there seem to be no positive criteria to distinguish it from the redness produced by inflammation. Under any circumstances, Mr. Porter assures us that he would hesitate to pronounce any appearance of redness on the internal surface of an artery, as indicative of inflammation, unless it were accompanied by other phenomena generally taken as the results or effects of this process on the arterial structure, such as thickening, friability, and softening, and some of which, it is probable, are developed with great rapidity, and at an early period.

Of the *causes* of arteritis nothing is certainly known.

The *symptoms* vary greatly in intensity. In some instances they are very obscure. In others, fever may run high, with severe pain in the course of the affected vessel. When this is the case the danger to the limb, and even to life itself may be very great. The artery is exceedingly painful, and like a chord to the touch; the pulsations may still continue, or be almost wholly obscured; the limb swells, becomes covered with phlyctenæ, and falls into gangrene, or, as has been observed by MM. Graves and Stokes, is for a time partially paralysed.

Mr. Porter divides arteritis into the healthy and the unhealthy—the former characterised by the effusion of coagulable lymph—the other by the absence of any such barrier to extension. The healthy is very generally the result of accident or injury: the unhealthy, as may be anticipated, is more frequently idiopathic.

*Consequences of Arteritis.*—These may be arranged in two classes—as it occasions alterations in the structure and functions of the vessel itself—or as it induces disease in other and remote parts.

“According to Cruveilhier, the essential character of commencing arteritis is the coagulation of the blood within it. This is observed in the healthy or adhesive inflammation occasioned by a division of, or the application of a ligature on, a vessel, when a coagulum is found to occupy the interspace between the ligature and the next collateral branch: but is still more remarkable in the unhealthy or spontaneous, where it is the most frequent cause of the gangrene that occurs in old persons. That coagulated blood is generally found after death within an inflamed artery is well established by dissection, but that this is the result of the inflammation is more than questionable. In a case that occurred in the Meath hospital,* and which was accompanied by the senile gangrene of the toes and feet; the femoral artery, which was greatly enlarged, was found, after death filled with a coagulum, but during life the blood unquestionably circulated through it, the motion imparting to the finger the sensation of a weak and indistinct thrill like that felt on touching the course of the urethra whilst the urine is passing. In another case treated in the same institution, where a ligature had been placed on the femoral artery, the segment below the chord exhibited the deepness of colour and the increased calibre of an inflamed vessel, but contained not a single particle of coagulum of any description, although there was no collateral branch within a considerable distance. Under these circumstances, and seeing that the aorta, at its arch or even before the arch is formed, is often the seat of inflammation, in which case the whole circulation should be stopped, it may be doubted whether the formation of a coagulum is an effect of inflammation during life; that which is found after the application of a ligature being the result of the stagnation of a small quantity of blood between it and the next collateral branch.” 380.

Mr. Porter acknowledges the difficulty of determining the exact nature of the primary appearances of arteritis. He thinks it may not be unfair to assume, that arteritis is ushered in by an increase of vascularity and of redness, followed, however, very speedily by an effusion of organizable coagulating lymph on the surface of the internal membrane; which, if the surfaces are laid together, will in a short space so glue and fasten them, as to close the vessel at that point.

He doubts, and so do we, the correctness of the opinion, that the obliteration of large tracts of arteries, such as that of the umbilical arteries and of the ductus arteriosus, is due to inflammation. Nothing can be more improbable. Mr. Porter attributes such obliteration to the operation of a general law in the animal economy, which tends to the closure of all canals, when no longer permeated by the substances for which they were designed to afford a passage.

*Symptoms.*—“ The very early symptoms of the unhealthy inflammation of

* “The phenomena and results of this case attracted considerable attention amongst the medical attendants and were particularly remarked by my friend and colleague Dr. William Stokes. The preparation is in the museum of the Park-street school.”

arteries are not known. In some instances, acute and severe pains have been experienced before the appearance of internal aneurisms, but in others there has been even more satisfactory evidence of the existence of the disease without the patient's complaining of any suffering: I can therefore only avail myself of examples in which the disease was fully formed, in order to describe its appearances and effects. When an opportunity is afforded of examining a vessel in this condition, and its coats are slit up longitudinally, the internal membrane appears to be of a bright crimson or carmine colour, varied with small spangle-like patches of a paler and more opaque tint. This vascularity is not confined to the lining membrane, for on removing this, the fibrous coat is seen also to be deeply stained. Between these, but more closely attached to the former (for it is removable with it,) is a deposition of a soft cheesy steatomatous material, which being seen through the lining membrane, gives it that patchy appearance already mentioned. An artery in this condition is remarkably increased in calibre; its properties, both of contractility and elasticity, are diminished, and it easily breaks down and tears, under an extending force that would have little or no effect on a vessel not the seat of disease. Probably in a very short time after the formation of this steatomatous deposit, ulceration commences in the lining membrane, which, proceeding from within, erodes the middle tunic to a greater or less extent of depth, and thus leads to the production of aneurism.

The symptoms that indicate the presence of arteritis, have not been clearly laid down, and moreover are such as might easily be overlooked, if not sought for by a careful and even rigid examination. It has been already stated that the previous occurrence of pain is not a satisfactory diagnostic. In the case of inflammation of the femoral artery already alluded to, Dupuytren stated, that a dull heavy pain had been previously felt in the iliac fossa of the same side, from whence it descended along the internal part of the thigh, then to the posterior part of the leg, and finally reached the sole of the foot and the toes. He also speaks of the patient having been annoyed by cramps, and in general, persons suffering from aneurism of the aorta accompanied by inflammation complain of pain in the back, stitches in the side, and spasms in different parts of the chest. Where gangrene is threatened, the temperature of the limb is diminished below the situation of the inflamed artery, and the coldness is felt to be increased in intensity as the examiner proceeds downwards, the sensibility being impaired exactly in the same proportion. On laying the finger over the course of the vessel, its pulsations are found to be weak, tremulous, and indistinct, the artery in its entire course appearing to be converted into a hard incompressible cord." 381.

Suppuration in an artery must be rare, but has been reported.

*Treatment.*—Acute and healthy arteritis should not, Mr. Porter thinks, be meddled with. He doubts whether the recommendation to treat the unhealthy form of arteritis on decided antiphlogistic principles be not rather too sweeping. He looks on it as analogous to erysipelas, believes that in each particular case the particular circumstances must be considered, is of opinion that the records of surgery offer nothing cheering or encouraging upon the subject, and in fact that fresh investigations are requisite.

Mr. Porter has satisfied himself that arteritis is one of the immediately exciting causes of aneurysm. He adds:—

" I have latterly investigated the history of every case that presented itself, and have come to the conclusion that intemperance, particularly in the use of ardent spirits, and repeated or ill-conducted courses of mercury, were most generally and most intimately connected with the production of arterial inflammation. This latter medicine is seldom used in great quantities, or for any protracted length of time, unless for the cure of syphilis, and hence many have entertained an opinion that arteritis might be a result of the operation of the venereal poison. In this supposition, I find Scarpa, Richerand, Corvisart, and to a certain extent, Mr. Hodgson, seem to coincide. Dupuytren, in examining arteritis in connexion with gangrene of the extremities, states that it is found almost always in persons that have indulged too freely in spirituous liquors, in stimulating food, and in those who have suffered from chronic diseases of the heart, or of the aortic valves or great vessels. Bouillaud, amongst the other causes that may produce inflammation generally, enumerates fatiguing and continued exercise, stimulating drinks taken in too great quantity, and the abuse of spirituous liquors as particularly conducing to arteritis." 382.

We would take the liberty of hinting to Mr. Porter that were he to compress his matter, arrange in a more natural order what he writes, and adopt a less rambling and a severer style, his observations would gain in force what they would lose in space. Those observations are in themselves so valuable, that we trust Mr. Porter will pursue his enquiries.

The Editor appends to these remarks of Mr. Porter's an account of the recent researches of M. Bizot, of Geneva, on the alterations of arteries.

"M. Bizot had observed but one form of alteration resulting from an acute attack of the internal lining of arteries. It showed itself upon the surface of this membrane in the form of an albuminous exudation, varying in thickness, as well as in consistence, and resembling jelly, being smooth, transparent, limpid, or rose-coloured. In some instances, the transparency of the adventitious membrane was such as to allow the pale colour of the artery to be distinguished beneath it. If a portion of the lining membrane be raised and torn off in the direction of the new deposit, the latter, behind which it is placed, and from which it may be separated, tears off with it. These exudations were in general confined to one spot, but they were also observed in scattered groups or patches of various sizes, and in some cases the transparent matter was so abundant as to fill up completely the calibre of the vessel. It is very frequently found at the orifice of the arteries arising from the arch of the aorta, of the cœliac, mesenteric, and renal arteries, and on the posterior surface of the aorta, obliterating partially or completely the orifices of the intercostals. Sometimes the albuminous spots appear puckered round their edges, which made Mr. Hodgson liken them to atrophied hernial sacs. When this matter exists in the form of isolated patches, it probably does not give rise to any general symptoms; but the case is just the reverse, when the morbid secretion is thrown out extensively on the internal surface of the large arteries. M. Bizot thinks that acute aortitis occurs in this way, and cites three examples observed by himself; the disease, in each case, terminating fatally. The symptoms observed were œdema accompanied by fever, but without any disturbance in the functions of the heart, or of the other organs essential to life; and these symptoms, he considers as indicating the extensive development of false membrane on the internal surface of the aorta." 383.

Mr. Costello quotes one of the cases at length. We must confess that we do not look on it as quite satisfactory. M. Bizot observes, in continuation, that the exudation of acute character may vary, however, from the description given above, being, in some instances, more intimately adherent to the lining membrane, more firm, and of a milky white. Sometimes these patches resemble the hard boiled white of egg, and present the hardness of cartilage; in the latter case, the lining membrane can no longer be found. That they severally commence in albuminous effusion cannot, however, be doubted, for if, while in the soft state, they be subjected to ebullition, the most transparent of these exudations become firm, opaque, and white. Some authors have described them as being developed between the internal and middle tunics, but this is erroneous, as may be shown, by peeling off the latter with great care: the shred stops or breaks off suddenly on arriving at the edge of the cartilaginous patches, and cannot be continued over its surface. In regard to these cartilaginous patches, another error is prevalent, viz. that they ultimately become osseous. Now if such transformation ever occurred, the stages of the transition must surely be detected, but this is not the case. Another peculiarity of these exudations is that they occur, most commonly, before or after the middle period of life.

M. Bizot next notices another form of alteration differing from the preceding in origin, in development, and in results. Alterations of this description may be observed even in subjects under puberty, presenting themselves singly or in groups, as small specks or dots of the size of a grain of sand, and of a whitish yellow colour, round or near the orifices of the coronary, innominate, carotid, subclavian, and intercostal arteries. They are not elevated upon the surface of the internal tunic, but are seen through it, as neither the transparency, nor the consistence of this membrane is in the slightest degree affected by their presence, at least, while in this, which may be considered, their *rudimentary* state. If a shred of the membrane be detached and torn towards these yellow spots, the spots peel off with it, being slightly adherent to its external surface; but if the same attempt be made, after the spots have become confluent, and formed a patch of some extent, the yellow substance then adheres partly to the outer side of the shred, and partly to the middle tunic; hence it is obvious, that these yellow spots are developed neither on the internal, nor on the middle tunic, but between both. When they attain a certain size, as they frequently do in old age, they undergo various transformations, of which, ulcerous, or rather atheromatous softening and ossification are the principal.

"The process of softening," continues M. Bizot, "presents various stages. In the first, the internal membrane covering the patch is still unaffected, but the internal fibres of the middle coat, on which it rests, are more or less altered according to the degree of advancement which the process has attained; these fibres are softened, and differ in colour, being of a brighter yellow than the sound parts of the same coat. The patch itself appears to be slightly elevated, but there is neither redness nor injected vessels in its vicinity.

In the second stage, the patches are elevated, like pustules of an irregular or rounded shape, on the surface of the aorta to the height of a line or two. The internal membrane is still thin, transparent, and unaltered. On pressing these eminences gently, the finger perceives the movement of a semi-fluid matter. If the pressure be increased, the lining membrane gives way, and a yellow matter escapes, which resembles healthy pus; sometimes this matter is either moist or dry, and as it were farinaceous, or the mass seems as if it consisted of a quantity of minute, thin, shining spangles, some of them of a silvery, some of a golden hue. The matter being evacuated, the pustule disappears, and the internal membrane is depressed in the place it occupied. On removing this membrane, the internal surface of the middle tunic is found ulcerated, softened, and reduced to a firm, moist, yellowish detritus; several of the laminæ of this tunic are thus altered, but the healthy structure still exists towards its most external layers.

In the third stage, the pustules having emptied themselves spontaneously, small irregular depressions, with edges slightly elevated, are observed. The surface of these depressions is smooth, but exhibits here and there fissures or cracks. On removing this internal membrane, the softened matter and the alteration of the middle tunic just described will be found. In some instances, the internal membrane has disappeared, and the blood is in contact with the middle tunic.

If we now compare these several lesions together, we must at once perceive that they are nothing more than degrees of one and the same alteration, which originated in the small point which was first observed between the internal and middle tunics, and which at a later period becoming fixed to the latter, passed through the stages above described." 386.

M. Bizot is of opinion that the succession of these changes proves to demonstration that the alterations described under the various names of spots, pustules, abscesses, atheromatous, and steatomatous productions, &c. have one common origin, to wit, the almost imperceptible spot, developed without any trace of inflammation between the innermost and middle tunics of the arterial tube. He gives to the cartilaginous patches, as our readers must have already observed, a totally different origin, *they* commencing in albuminous effusion in or on the internal membrane.

*Osseous Transformation.*—Sometimes, proceeds M. Bizot, a hard semi-transparent point, of the same colour as the patch itself, forms in its centre, and increases slowly, more in extent than thickness, till it alters the structure of the middle tunic, as has been already described. The surface in contact with the lining membrane increases less rapidly, and a long period elapses, even when the osseous disk is of some size, before any remarkable change can be observed in this membrane. In some cases, however, it seems to be slightly thickened by a layer of albumen spread over the concretion. Ultimately, even this membrane is destroyed by the progress of the ossification, which then is in immediate contact with the blood. The yellow soft matter always surrounds the concretion, but no fibres can be discovered where they are in contact, the fibrous structure of the middle coat can only be detected by proceeding from the circumference of the concretion towards the sound portion of the artery. It is in the advanced period of life that these concretions are most commonly formed. But they have also been discovered in subjects of tender age. Mr. Hodgson mentions a case observed by Mr. Young, in which the temporal artery of a child only fifteen months old was found ossified, and Wilson met an instance of ossification in the aorta of a child only three years old. Bichat supposed that ossification never occurred in vessels through which black blood only circulated, and consequently that these concretions were not observed in the pulmonary artery. This, however, is erroneous, for, though rare, they occur.

Although the cartilaginous concretions will exist simultaneously with the ulcerous softening of the middle tunic, and with the osseous concretions, still the former is not transformed into the latter. They have, so M. Bizot tells us, the separate origin that he describes.

*Alterations peculiar to the Arteries of the Limbs.*—Of these M. Bizot gives the following account. They first appear, he says, as minute stains; they adhere to the middle tunic, and cannot be torn off with the lining membrane. Becoming multiplied they extend round the artery like a zone, and seem to be slightly depressed below the general surface of the cavity of the vessel; their adhesion to the middle tunic is so firm, that they cannot be separated from it, like the yellow patches in the large arteries, when we tear off a shred of the lining membrane. The fibres of the middle tunic at these points appear to be yellower, harder, thinner, less pliant, and more resisting. The transformation commencing immediately beneath the lining membrane, which it never affects, extends outwards through the layers of the middle tunic. The fibres at length are changed into depressed osseous, lines, which uniting, complete the entire circle of the vessel, and extend to various lengths upwards and downwards along its course. When the ossification of the middle tunic is complete, the artery is reduced to the condition of an inert tube, the external and internal tunics which are healthy merely covering both its surfaces. In undergoing this transformation, the middle tunic is diminished in thickness, so that the diameter of the artery becomes actually larger. This account will be observed to differ in several respects from that which has been given of the progress of the atheromatous deposit in the larger vessels. Yet the two changes may appear together.

Mr. Costello makes a curious statement, which we confess takes us rather by surprise.

"In the course of M. Bizot's researches, he was struck with the fact, that whenever he found a lesion in an artery on one side of the body, the same kind of lesion would in all probability be found at the same point of the corresponding artery of the opposite side. With some exceptions, which might be easily accounted for, this symmetrical development of lesions was so constant, that he does not hesitate to characterize it as an invariable law in morbid anatomy." 388.

We will not say that it is not so, but we own that we doubt if this be true, au pied de la lettre. On the whole, however, M. Bizot's observations deserve attention, and should be put to the test of accurate investigation.

The articles "Traumatic Arthritis," and "Articulations," by J. Blackburn, are written with ability and judgment. From "Asphyxia," by Mr. Bennett Lucas, we are tempted to extract some criticisms on the directions promulgated by the Royal Humane Society for the recovery of persons apparently dead by drowning.

The Society direct, among other things, the application of external heat, and they particularly laud the employment, where it is possible, of the warm-bath. On this Mr. Lucas remarks:—

"Were the directions here recommended to be implicitly obeyed, the enfeebled vital actions would be in danger of extinction, and the 'apparent death' would soon become real. The temperature of the apartment in which the body is laid, should in general not be higher than 75° or 80°; and Dr. Edwards even recommends in some cases that it should be lower than this. The advantage to be derived from exposing the surface of the body to the vivifying influence of the air is altogether lost sight of, when the body is recommended to be 're-clothed,' 'to be wrapt in blankets wrung out with hot water,' or to be immersed in a 'warm-bath.' Where heat is demanded, its application is therefore preferable in the radiant form, and when *conducted* heat is called for, it should be so partial in its application, as to interfere but little with the exposure of the surface to the 'vivifying influence' of the air. The employment of heat in the radient form also gives us the opportunity of applying friction and other excitants to the body, and does not interfere with our endeavours to restore life by artificial respiration.

The body should therefore be placed before the fire, or in the sunshine, and bottles of warm water, or warm bricks, applied to limited portions of the cutaneous surface, such as the soles of the feet, or the arm-pits. A proper temperature also may be applied to the spine and back by means of the tin mattrasses filled with warm water, such as were recently used during the prevalence of cholera, in several of the hospitals. By these proceedings the influence of the air on the skin, in rousing the action of the capillaries, and imparting oxygen to the blood, is uninterfered with, whilst friction, the application of stimuli, and, above all, artificial respiration, can be advantageously resorted to.

We have been thus particular in detailing the researches of Dr. Edwards and their application to the human being when in a state of asphyxia, in consequence of the directions of the Humame Society being sanctioned by the authority of others who have treated of this subject. And no matter how great the caution exercised in avoiding the too sudden application of heat, the circumstances of the case must be very peculiar indeed which should warrant us in raising it beyond what we have already recommended, much less to the height of '98° or 100°,' 430.

We cannot help suspecting that the "vivifying influence of the air" has been pitched rather too high, since Dr. Edwards' experiments. Yet, notwithstanding, we are inclined to agree with those who reprobate the warm bath. Independently of its interfering with the action of the skin, the body is a good deal pulled and hauled about in getting it into and out of the bath, and delay and interference with other means result. Then, artificial respiration is managed less easily in the bath than out of it. Taking all these circumstances into the account, (and we speak from practical experience,) we look upon Mr. Lucas's strictures as not altogether unjust, and are of opinion that the Society would do well to revise their recommendations in this particular.

